# Small Bowel Perforation Due to Rare Metastasis From Stage IV Lung Adenocarcinoma

**DOI:** 10.7759/cureus.29551

**Published:** 2022-09-25

**Authors:** Francesco Sautto, Sarah Tinsley, Vladimir Neychev

**Affiliations:** 1 Surgery, University of Central Florida College of Medicine, Orlando, USA; 2 Pathology, University of Central Florida (UCF) HCA Lake Nona Hospital, Orlando, USA

**Keywords:** metastatic non-small cell lung cancer, small bowel perforation, acute peritonitis, small bowel metastasis, metastatic adenocarcinoma of lung

## Abstract

Lung adenocarcinoma commonly metastasizes to the lymph nodes, bone, nervous system, liver, respiratory system, and adrenal gland. Metastasis to the small bowel is extremely rare and the literature deals mostly with few published case reports. We present a case of a 70-year-old male with a previous history of stage IV lung adenocarcinoma with brain and liver metastases treated with radiotherapy and chemotherapy. He presented to the emergency department with peritonitis and sepsis due to bowel perforation. He underwent an emergency exploratory laparotomy and small bowel resection. Surgical pathology revealed metastatic lung adenocarcinoma as the cause of bowel perforation. He tolerated and recovered from surgery well and was discharged on postoperative day 7. On one-month follow-up as an outpatient, he was doing well and had no complications or complaints from his recent surgery.

## Introduction

The number one cause of cancer mortality within the United States is lung cancer [1]. Given that cancer can present itself at more advanced stages, physicians must know both common sites of metastasis as well as less common sites. The most common sites for lung cancer are lymph nodes (48%), liver (45%), adrenal gland (41%), bone (31%), and brain (25%) with a less common occurrence being the gastrointestinal tract (GI) (11.9%) [2,3]. Reports have previously shown that the small bowel is the most frequently noted gastrointestinal site of metastasis (10.67%) [2,3] with many cases presenting with either obstruction or perforation [4]. While there are case reports describing similar scenarios in the literature [5,6], the incidence of small bowel metastasis from lung cancer remains low [7]. Yet, it is important that physicians caring for patients with metastatic lung cancer presenting with acute peritonitis or abdominal symptoms keep a possible GI metastasis in their differential.

We report a case of a man with late-stage lung adenocarcinoma presenting with clinical and para-clinical symptoms of acute peritonitis secondary to perforation from a metastatic lesion. We also provide a brief review of case reports and retrospective chart review findings to provide insight into the case presented.

## Case presentation

**Figure 1:**
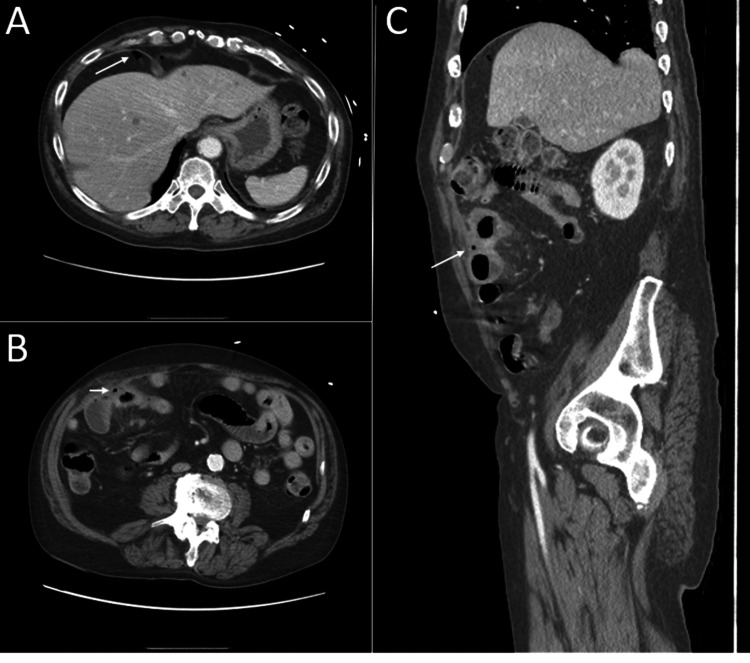
CT of the abdomen and pelvis with IV contrast (A) Axial view showing free air pocket under the right diaphragm. (B) Axial view showing small extraluminal air pocket and fluid collection between small bowel loops and wall of the right lower quadrant. (C) Sagittal view showing pocket of free air and fluid collection.

**Figure 2:**
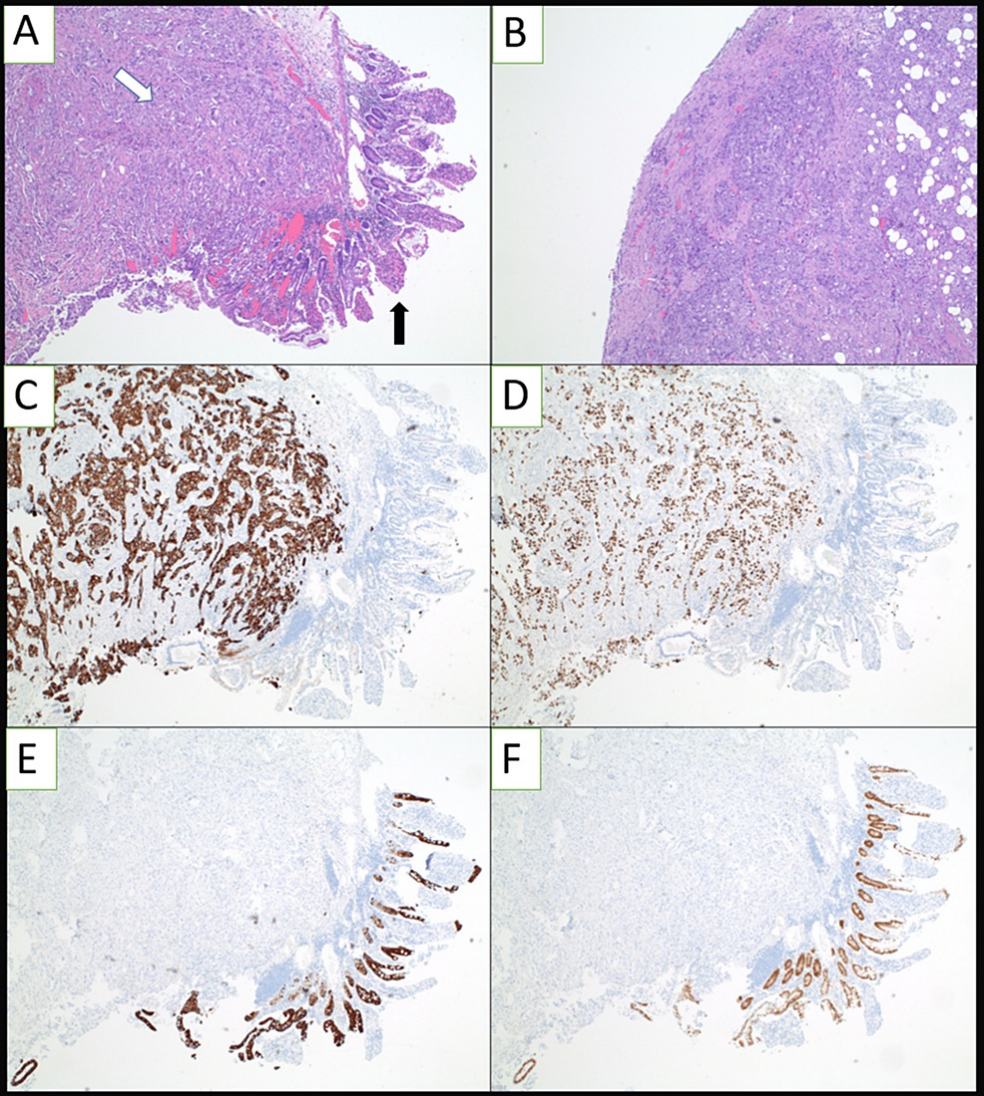
Hematoxylin and eosin stain, and immunohistochemistry of tumor sample (A) Low power visualization of tumor sample (white arrow) with residual small intestine (black arrow). (B) Low power visualization of tumor with positive serosa. (C) Immunohistochemistry study positive for CK7 on tumor. (D) Immunohistochemistry study positive for TTF1 on tumor. (E) Immunohistochemistry study positive for CK20 on residual small intestine. (F) Immunohistochemistry study positive for CDX-2 on residual small intestine.

A 70-year-old male with a past medical history of hypertension and hyperlipidemia was diagnosed two months ago with Stage IV Lung Adenocarcinoma of the right upper lung lobe metastatic to the brain and liver, after a witnessed fall incident. He had finished 1 course of cranial radiotherapy for a solitary brain mass, and one cycle of systemic chemotherapy, a week before he presented to the emergency department (ED) with progressively worsening, diffuse abdominal pain over two days. He did not report any significant abdominal symptoms or changes in bowel movements prior to the current onset. The workup in the ED included a complete blood cell count showing a WBC count of 28×10^3^/µL and platelet count of 252x10^3^/µL, and computed tomography (CT) chest/abdomen which revealed free peritoneal air with an interloop abscess at the distal jejunum concerning for perforation (Figures 1A-1C). The decision was made to perform an emergent exploratory laparotomy rather than a laparoscopy due to signs of acute peritonitis. During the operation, a small bowel perforation measuring approximately 0.5 cm at the level of distal jejunum-proximal ileum with interloop juxta mural abscess was identified concerning metastasis. Intraoperative findings corresponded to an ulcerated tumor mass involving most of the bowel circumference and measuring 3 cm x 3.5 cm. Approximately 30 cm of small bowel 15 cm proximal and 15 cm distal to the perforation site was resected and sent to pathology as a permanent specimen, along with portions of the mesentery with grossly enlarged lymph nodes. The continuity of the GI tract was reinstituted with a side-to-side functional end-to-end anastomosis and the mesenteric defect was closed. The small intestine was run from the ligament of Treitz to the ileocecal valve twice and no further pathologies were identified. The peritoneal cavity lavage with warm normal saline was performed. The patient tolerated the procedure well, was extubated, and was sent to the PACU in stable condition. The patient’s postoperative course was complicated by paroxysmal atrial fibrillation that was managed by Cardiology. Otherwise, his recovery from surgery progressed as expected and the patient was discharged on day 7 post-operatively with plans to continue systemic chemotherapy as recommended by his oncologist. The pathology report of the small bowel resection revealed metastatic adenocarcinoma involving 9/9 lymph nodes and negative margins. Immunohistochemistry (IHC) revealed that the mass was CK7 and TTF-1 positive, confirming a diagnosis of metastasis from lung adenocarcinoma (Figures 2A-2F). The patient was seen for outpatient follow-up after one month and doing well with no complications or complaints related to the operation. He plans to continue with systemic chemotherapy.

## Discussion

Non-small cell lung cancer is more common than small cell lung cancer and comes in three main types, adenocarcinoma, squamous cell carcinoma, and large cell carcinoma, with advanced cases presenting with metastasis to the brain, lymph nodes, adrenals, or bone but rarely to the gastrointestinal tract [2]. However, it is questionable as to whether the scarcity of GI metastasis from lung cancer is due to it being undiagnosed in asymptomatic presentations versus presentations of acute peritonitis [6]. There still remain cases of GI metastasis in the literature [5,6] and thus it is key for physicians caring for patients with lung cancer to be aware of abdominal symptoms that may be significant for a metastatic lesion. To assess our case compared to current literature, we performed a Medline/Pubmed search for “small bowel metastasis” or “gastrointestinal metastasis” and “pulmonary carcinoma” for literature reviews on gastrointestinal metastasis of lung cancer, utilizing reference sections to search for additional reviews as needed. The results and details of the literature reviews can be found in Table 1. 

**Table 1:** A collection of literature and chart reviews on gastrointestinal metastasis of lung cancer.

Author	Year	Country	Chart Review or Lit Review	Number of patients with lung cancer	Number with GI mets	Clinical Symptoms (n)	Surgery (n)	Type of Lung Cancer (n)	Site of Metastasis for those with GI mets (n)	Associated with other metastatic sites	Patient survival after surgery
McNeil [2]	1987	USA	Chart Review	431	46	Perforation (14/15) Obstruction (1/15)	Laparotomy	Squamous Cell (32.6%) Adenocarcinoma (28.3%) Small Cell (13.0%) Large Cell (26.1%)	Small Bowel (46/431)	4.8 sites average Lymph nodes, adrenal gland, liver, kidney	All <4 months
Berger, A [8]	1999	France	Chart Review	1544	7	Acute peritonitis (2) Obstruction (3) GI Bleeding (2)	Intestinal Resection (6) Bypass (1)	Squamous Cell (3) Large cell (2) Adenosquamous (1) Adenocarcinoma (1)	Jejunum (29%) Ileum (43%) Both (28%)	Unspecified	8 months (6) Alive (1)
Garwood, R [5]	2004	USA	Literature Review	98	98	Perforation (98)		Adenocarcinoma (23.7%) Squamous Cell (22.7%) Large Cell (20.6%) Small Cell (19.6%)	Jejunum (53%) Ileum (28%) Both (4%) No specific site (13%)	Unspecified	66 days mean survival 30 days (50%) >1 year (2%)
Yoshimoto, A [3]	2006	Japan	Chart Review	470	56	Of those presenting with life threatening metastasis (n=12): Perforation (33%) Appendicitis (17%) GI Bleeding (33%) Ileus (17%) Intussusception (8%)	Unspecified	Adenocarcinoma (44.6%) Large Cell (21.4%) Small Cell (17.9%) Squamous (14.3%) Adenosquamous (1.8%)	Small Intestine (68%) Gastric (43%) Large Intestine (38%) Multiple (52%)	Yes, Liver, Adrenal, Kidney, Abdominal lymph node	Unspecified
Goh, B [9]	2007	Singapore	Chart Review	9	9	Obstruction (22%) Hemorrhage (33%) Intussusception (33%) Perforation (11%)	Small bowel resection with primary anastamosis (55%) Subtotal gastrectomy with extended right hemicolectomy (11%) Gastrojejunostomy (11%) Right hemicolectomy (11%) Ulcerectomy (11%)	Squamous Cell (33%) Large Cell (33%) Adenocarcinoma (11%) Pleomorphic (11%) Pleomorphic with adenocarcinoma (11%)	Ileum (33%) Jejunum (33%) Cecum (11%) Duodenum (22%) Stomach (22%)	Yes, brain, liver	89% discharged with median survival 6 months 50% >6 months 1 Alive
Po-Chu, Lee [4]	2011	Taiwan	Chart Review	8159	21	Perforation (14%) Hemorrhage (52%) Illeus (9.5%) Obstruction (14%) Abdominal Pain (14%) Tenesmus (9.5%)	Segmental Resection (24%) Right Hemicolectomy (14%) Wedge resection of stomach (4.7%) Proximal Gastrectomy (4.7%)	Adenocarcinoma (57%) Squamous Cell (24%) Pleomorphic (4.7%) Small Cell (4.7%) Undifferentiated (4.7%) Adenosquamous (4.7%)	Small Bowel (29%) Stomach (38%) Duodenum (14%) Colon (14%) Multiple (9.5%)	Yes, brain, bone, liver, adrenal gland, gall bladder, panceas, peritoneal seeding, ovary, uterus	1-3 months (62%) < 1 month (19%) >3 months (19%)
Jian-Zhong, D [6]	2014	China	Literature Review	100	100	Perforation (46%) Obstruction (35%) GI Bleeding (14%)	Exploratory laparotomy with resection and end-to-end anastamosis	Large Cell (32%) Squamous Cell (27%) Adenocarcinoma (23%) Adenosquamous (3%) Carcinosarcoma (2%)	Only small bowel metastases included	Yes (70%) Brain, adrenal gland, bone, liver	Median 2.3 months
Taira, N [7]	2017	Japan	Chart Review	2066	7	Perforation (2) Gastric Bleeding (2) Intussusception (1) Acute Appendicitis (1) Colonoscopy (1)	Emergency Laparotomy (4) Gastroendoscopy (2) Colonoscopy (1)	Adenocarcinoma (4) Large Cell (1) Pleomorphic (2)	Small Bowel (3) Gastric (2) Large bowel (1) Appendix (1)	Brain (2) Bone (2) Skin (1) Adrenal (1)	6 months (1) 4 months (1) 5 months (1) 2 months (1) 3 months (1) 21 days (1)

The case presented was elucidated by a combination of imaging along with clinical and para-clinical symptoms whereby the finding of interloop jejunal abscess with progressive abdominal pain and free peritoneal air was suspicious for metastasis and perforation gave the patient’s history of multiple metastases to the brain and liver from lung adenocarcinoma. A review of the literature found that the median time between lung cancer diagnosis and GI metastasis is three months [4], which fits within the timeframe of our patient’s presentation. Our diagnosis was confirmed with exploratory laparotomy, which was necessary given the evidence for perforation, yet a CT abdomen done one month prior to this event during initial staging had not revealed metastasis to the small bowel. The use of CT imaging is adequate to confirm perforation or abscess in the setting of an acute abdomen though it is difficult to distinguish small metastatic lesions within the small bowel [10], and PET/CT, which can theoretically show increased uptake to metabolically active lesions, have not shown to demonstrate a diagnostic advantage over CT alone [11]. IHC for the specimen showed CK7 and TTF-1 positively which is in line with Lung Adenocarcinoma, which has been shown in several studies to be the leading type of lung cancer in small bowel metastases [3-5,7]; however, other studies may indicate that either squamous cell carcinoma [2,8,9] or large cell carcinoma [6,9] is more likely. This is further supported by TTF-1 having been shown to appear in 89% of metastatic lung adenocarcinoma cases and CK7 having been shown to appear in all cases of lung cancer [11]. One report indicated that large cell carcinoma has an overall 3.5 times greater rate of gastrointestinal metastasis and accounted for 30% of those metastases [6].

Without evidence of perforation, it may have been vastly difficult to be able to diagnose metastasis in the patient described. Perforation itself is a poor prognostic indicator in the realm of intestinal metastasis with lung cancer [6]. While our patient underwent a round of chemotherapy a few days prior to presentation at our hospital, there is inconsistent data to show an increased incidence of perforation, though a majority (75.5%) received either single or combined therapy 2 days to 4 years prior to a perforating event [5]. In similar situations, it has been found that aggressive surgical intervention can provide effective palliation for lung cancer patients with acute abdomen secondary to gastrointestinal metastasis, though in that study all those who survived more than 6 months did not have extrapulmonary spread outside of the gastrointestinal tract [9], unlike our patient presenting with both brain and liver metastases. Several different reviews suggest that perforation may be a leading presentation for pulmonary metastasis to the small bowel as compared to obstruction, intussusception, or gastrointestinal bleeding [3,4,6], though other reviews show a more mixed incidence of presentations [7-9].

Other reviews indicated that all small bowel and colon metastases required surgery to relieve either obstruction or perforation [3,4]. In addition to our case, this highlights the importance of being able to identify small bowel metastasis prior to the onset of acute abdomen. Possible approaches include video capsule endoscopy (VCE) which has been shown to be able to diagnose small bowel tumors in places not easily reachable by traditional endoscopic methods, along with regular colonoscopies for colon metastases [12,13]. Our patient had a colonoscopy with non-significant findings two years prior to his cancer diagnosis, and a CT abdomen one month prior to the perforation event, which may indicate that such screening tests may not be well equipped to diagnose metastasis from lung cancer.

Overall, previous reviews have been mixed on what the most common gastrointestinal site is for lung cancer metastasis with leading sites being the jejunum and ileum, followed by the duodenum and the cecum. The exploratory laparotomy in this case found the perforation and metastasis at the proximal ileum-distal jejunum given that it manifested as a loop abscess, making the exact location difficult to define.

Our patient had a post-operative course complicated with paroxysmal atrial fibrillation but otherwise was able to be discharged on postoperative day 7 and found to be doing well on a one-month outpatient follow-up. Survival means post-surgical intervention, in one report was six months, though those patients were specifically selected and did not have any other extrapulmonary metastasis [11]. The literature is mixed in survival rates, with some noting median rates of 2.3 months or a greater majority of patients surviving until one to three months [4,6].

## Conclusions

To summarize, our case is one of a number of reports of lung cancer spreading to the small bowel, resulting in perforation and subsequent necessitation for exploratory laparotomy. This adds to the growing literature on this occurrence and could indicate that the prevalence of such metastasis may be higher than expected with most only being ascertained at the point of perforation. Data might also need to be collected on whether chemotherapy treatment on patients with lung cancer and unknown GI metastasis may allow for risk factors that induce a perforating event.
